# Supplementary figures and images for: Proteomic Analysis of Exosomes Secreted from Human Alpha-1 Antitrypsin Overexpressing Mesenchymal Stromal Cells
Source: Biology (Basel). 2021 Dec 21;11(1):9. doi: 10.3390/biology11010009 (PMC8773149; doi:10.3390/biology11010009)

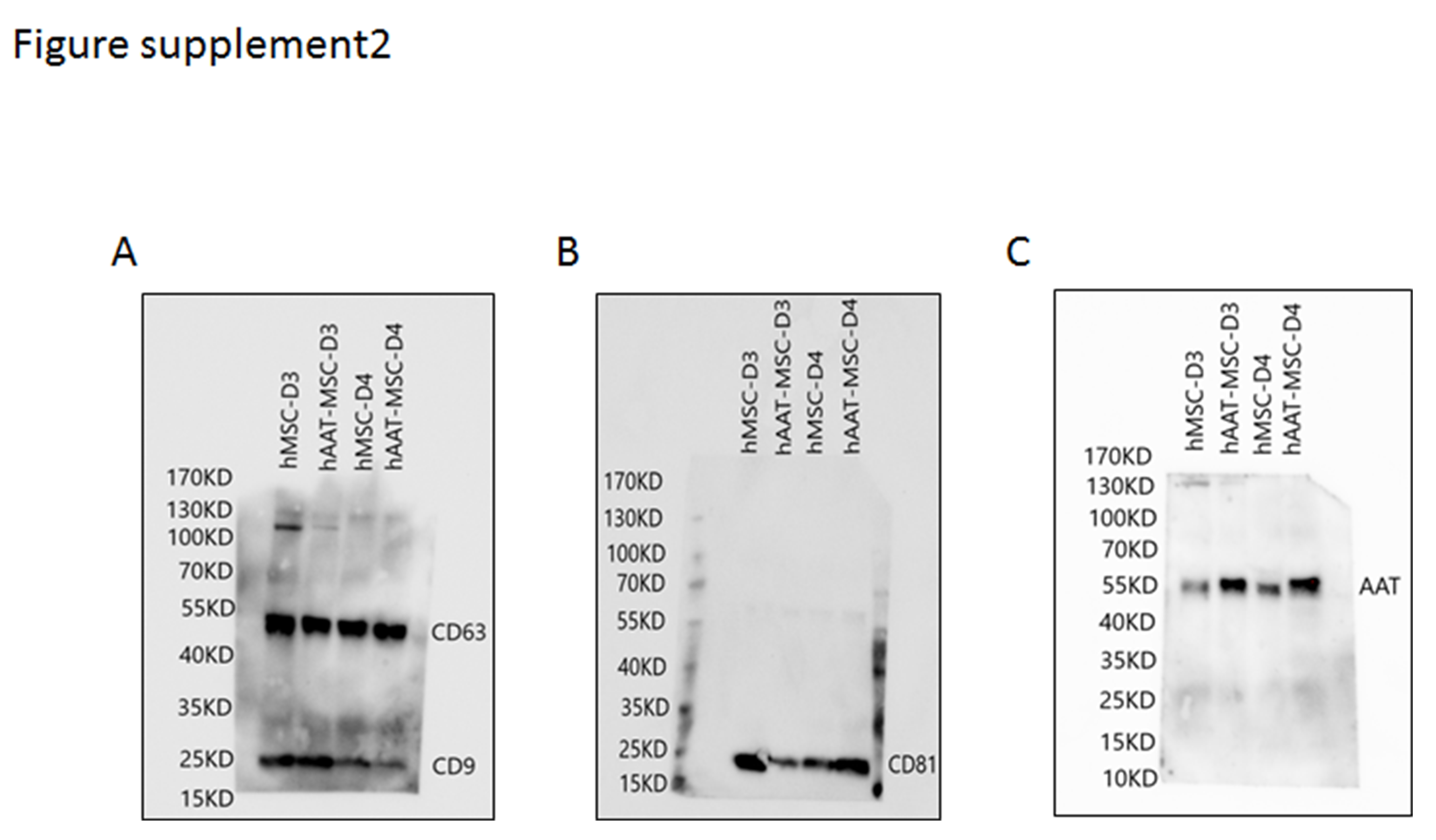

Supplement: Supplementary file 1 [file biology-11-00009-s001.zip › FigureS2.tif]
